# The Vanishing Clinical Value of PD-L1 Status as a Predictive Biomarker in the First-Line Treatment of Urothelial Carcinoma of the Bladder

**DOI:** 10.3390/cancers16081536

**Published:** 2024-04-17

**Authors:** Alexander Tamalunas, Can Aydogdu, Lena M. Unterrainer, Melanie Schott, Severin Rodler, Stephan Ledderose, Gerald B. Schulz, Christian G. Stief, Jozefina Casuscelli

**Affiliations:** 1 Department of Urology, LMU University Hospital, LMU Munich, 81377 Munich, Germany; can.aydogdu@med.uni-muenchen.de (C.A.); melanie.schott@med.uni-muenchen.de (M.S.); severin.rodler@med.uni-muenchen.de (S.R.); gerald.schulz@med.uni-muenchen.de (G.B.S.); christian.stief@med.uni-muenchen.de (C.G.S.); jozefina.casuscelli@med.uni-muenchen.de (J.C.); 2 Department of Nuclear Medicine, LMU University Hospital, LMU Munich, 81377 Munich, Germany; lena.unterrainer@med.uni-muenchen.de; 3 Department of Pathology, LMU University Hospital, LMU Munich, 81377 Munich, Germany; 4 Comprehensive Cancer Center (CCC Munich LMU), LMU University Hospital, 81377 Munich, Germany

**Keywords:** bladder cancer, systemic treatment, biomarker, PD-L1, oncology, urology

## Abstract

**Simple Summary:**

Bladder cancer is the sixth most common cancer in the world. With 73 years, bladder cancer has the highest age-at-diagnosis of all cancers. While the surgical removal of the bladder remains the standard-of-care treatment for advanced muscle-invasive disease, around half of patients still metastasize. Then, platinum-based chemotherapy is recommended as the first-line treatment. However, only about half of those patients are eligible to receive chemotherapy, and novel immune-checkpoint inhibitors are restricted to biomarker (PD-L1)-positive patients. In our study, we demonstrate that PD-L1-positive patients show slower progression. However, there is no benefit in overall survival, emphasizing the need for novel and more reliable biomarkers in the future.

**Abstract:**

Background: Our study endeavors to elucidate the clinical implications of PD-L1 positivity in individuals afflicted with advanced urothelial carcinoma of the bladder (UCB). Methods: Patients with advanced UCB were prospectively enrolled following a radical cystectomy (RC) performed within January 2017 to December 2022 at our tertiary referral center. The clinical outcome, defined as the progression-free survival (PFS) and overall survival (OS) on systemic treatment, was analyzed using an χ^2^-test, Mann–Whitney U-test, the Kaplan–Meier method, and a log-rank test. Results: A total of 648 patients were included following an RC performed within January 2017 to December 2022. Their PD-L1 status was analyzed with the primary PD-L1-specific antibody (clone SP263, Ventana) and defined both by the CPS and IC-score in 282 patients (43.5%) with a high risk (pT3–pT4 and/or lymph node involvement) or metastatic UCB. While the median PFS was significantly prolonged 5-fold in PD-L1+ patients, we found no difference in OS, regardless of PD-L1 status, or treatment regimen. Conclusions: While PD-L1 positivity indicates prolonged PFS, the presence of PD-L1 does not influence OS rates, suggesting its limited usefulness as a prognostic biomarker in bladder cancer. However, the positive correlation between an PD-L1 status and a sustained response to ICI treatments indicates its potential role as a predictive biomarker. Further research is required to understand how the predictive value of PD-L1 positivity may extend to the use of ICIs in combination with antibody-drug conjugates.

## 1. Introduction

In the United States alone, urothelial carcinoma of the bladder (UCB) is estimated to be the sixth most common cancer, with over 82,000 newly diagnosed cases and approximately 18,000 disease-associated deaths per year [1]. At the time of diagnosis, a quarter of patients already exhibit muscle invasive bladder cancer (MIBC), with rates remaining unchanged over the past decade according to data from the Surveillance, Epidemiology, and End Results (SEERs) registry [2,3,4]. Radical cystectomy (RC) remains the primary curative treatment for non-metastasized MIBC [5,6]. However, about half of patients metastasize following curative RC [7]. Platinum-based chemotherapy is then the standard-of-care for the metastasized urothelial carcinoma of the bladder (mUC), with an overall survival (OS) for cisplatin-based chemotherapy being 9–15 months [8,9]. With 73 years, UCB has the highest median age-at-diagnosis of all cancers, and patients’ ages and concomitant comorbidities naturally limit cisplatin eligibility [8,10,11]. Unfortunately, the response to alternative treatment regimens is limited [10].

With the approval of the immune checkpoint inhibitors (ICIs) atezolizumab and pembrolizumab by the *Food and Drug Administration* (FDA) and the *European Medicines Agency* (EMA), promising new options for cisplatin-ineligible patients in first-line (1-L) therapy have become available as of 2017 [12,13]. While pembrolizumab targets programmed death-1 (PD-1), atezolizumab targets programmed cell death 1 ligand-1 (PD-L1) [12,13]. The interaction of PD-1 on T cells with its ligand PD-L1 on tumor and immune cells limits T-cell-mediated immune response, assisting in the immune system escape by the tumor [14,15]. PD-L1 positivity can be expressed as the *Immune Cell* (IC) or *Combined Positive Score* (CPS) by immunohistochemical staining, and the plasticity of PD-L1 expression in various tumor entities is still up for debate [16,17,18]. In addition, it has been suggested that PD-L1 expression may be associated with certain clinico-pathological features and clinical outcomes [19,20]. With multiple tumor entities showing PD-L1 expression, including UCB, and due to the disproportionately long-lasting efficacy in a subgroup of patients receiving ICI, predictive biomarkers for patients with UCB have been discussed [18,21,22,23].

Information on the immunohistochemical expression of PD-L1 was gathered during trials, which initially lead to the approval of atezolizumab and pembrolizumab in 2017. However, the efficacy of these agents was higher in the subgroup of patients expressing PD-L1, eventually leading to the FDA and EMA restricting 1-L treatment to PD-L1-positive patients in 2018 [24,25]. ICIs were further evaluated for patients eligible for chemotherapy, where combinations of ICIs demonstrated slightly superior oncological benefits compared to those of chemotherapy alone, although the efficacy of ICI monotherapy did not surpass that of chemotherapy [26,27,28]. In these clinical studies, PD-L1, as a sole biomarker, lacked sufficient robustness and reproducibility.

In our study, we sought to characterize PD-L1’s expression and its correlation with clinico-pathological features, immune-related adverse events, progression-free survival (PFS) and overall survival (OS) in a large series of patients with UCB, including patients who developed metastatic disease and were subsequently treated with ICIs or platinum-based chemotherapy in the 1-L setting.

## 2. Materials and Methods

### 2.1. Clinical Cohort

**Figure 1:**
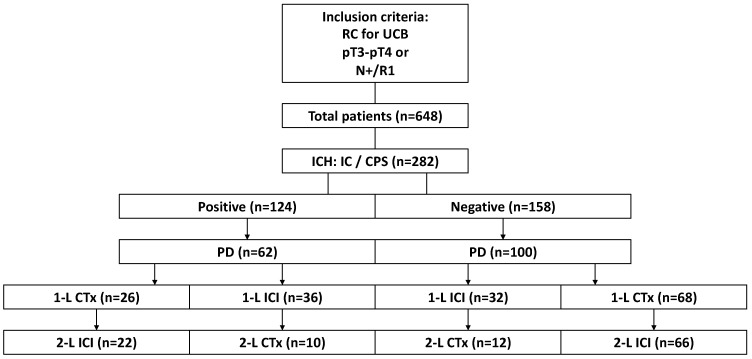
Patient cohort and study design. Shown are the inclusion criteria of patients with advanced disease: a radical cystectomy (RC) for urothelial carcinoma of the bladder (UCB) presenting with tumor stage ≥ pT3, positive lymph nodes (N+) or a positive resection margin (R1). ICH = immunohistochemistry; PD-L1 = programmed death 1 ligand 1; 1-L = first line treatment; 2-L = second line treatment; IC = immune cell; CPS = combined positive score; CI = confidence interval; CTx = chemotherapy; ICI = immune checkpoint inhibitor; PD = progressive disease.

We prospectively enrolled 648 patients with advanced UCB, who a received curative RC at our tertiary referral center within the time period of January 2017 to December 2022. Our research was carried out in accordance with the Declaration of Helsinki of the World Medical Association and has been approved by the ethics committee of Ludwig Maximilians University, Munich, Germany. Informed consent was obtained from all patients. Inclusion criteria are outlined in Figure 1. However, only two patients in our cohort received Atezolizumab for 1-L treatment, and only four patients received any ICIs other than pembrolizumab in 2-L treatment. Thus, only patients receiving pembrolizumab as an ICI treatment were included into our final analysis. A computerized database containing clinical and pathological information, as well as follow-up information, was created specifically for this study. An expert pathological review regarding grading and staging parameters was conducted for all histopathological samples. Histological classification was based on the 2004 and 2016 WHO/International Society of Urological Pathology grading systems [29]. Clinical outcomes were defined as progression-free survival (PFS) and overall survival (OS) on systemic treatment. All data were irreversibly anonymized before analysis, and institutional review board approval was obtained before data acquisition.

### 2.2. Immunohistochemistry and Scoring of Protein Expression

Immunohistochemical (IHC) staining was conducted to assess programmed death-ligand 1 (PD-L1) expression using formalin-fixed paraffin-embedded (FFPE) blocks obtained from RC specimens at the Department of Pathology, University Clinic Aachen, Germany. The staining protocol used a Ventana Benchmark XT autostainer (Ventana Medical Systems, Oro Valley, AZ, USA) following the manufacturer’s instructions. Heat-induced epitope retrieval was achieved by treating with Cell Conditioning 1 (CC1, pH 8.4) (Ventana Medical Systems, Oro Valley, AZ, USA) for 64 min, followed by incubation with a primary PD-L1-specific antibody (clone SP263, Ventana, ready-to-use, Ventana Medical Systems, Oro Valley, AZ, USA) for 16 min at 36 °C. Detection was carried out using the Ventana OptiView DAB IHC Detection Kit. Subsequently, all slides were counterstained with hematoxylin (Vector Laboratories, Burlingame, CA, USA). The evaluation of PD-L1 expression included two parameters: the combined positive score (CPS) and the immune cell (IC) score. The CPS was calculated by dividing the number of PD-L1-positive cells (including tumor cells, lymphocytes, and macrophages) by the total number of viable tumor cells, then multiplying by 100. The IC score represented the percentage of the tumor area covered by PD-L1-positive immune cells and was categorized as follows: IC 0: <1%, IC 1: 1–5%, IC 2: 5–10%, and IC 3: >10% [30]. Regions of necrosis within the tumor fields were excluded from the assessment. In accordance with the established criteria and guidelines for PD-L1 scoring in urothelial carcinoma, specimens with a CPS ≥ 10 and/or an IC score ≥ 2 were classified as PD-L1 positive. Importantly, PD-L1 scoring was conducted in a blinded manner, without access to patient identifiers or clinical outcomes.

### 2.3. Statistical Analysis

Statistical analysis was performed using SPSS V29.0.0 software (IBM SPSS Statistics, Version 29.0.0 Armonk, NY, USA). The primary endpoint was PFS with disease progression determined by the attending physician. Additionally, we analyzed OS as a secondary endpoint, which was determined by the attending physician or according to an official death certificate. The final analysis of follow-up was performed in December 2023. Categorical variables underwent comparison through the Pearson χ^2^-test, while continuous variables were evaluated using the Mann–Whitney U-test. Progression-free survival (PFS) and overall survival (OS) were depicted using Kaplan–Meier methodology, with between-group differences assessed via the log-rank test. All reported *p*-values were two-sided, with statistical significance determined at *p* < 0.05.

## 3. Results

### 3.1. Patient Characteristics

We prospectively included 648 patients with advanced UCB, who received a curative RC within January 2017 to December 2022 (Figure 1). PD-L1 status was analyzed using a primary PD-L1-specific antibody (clone SP263, Ventana) and described as both the CPS and IC score in 282 patients (43.5%) with a high risk (pT3–pT4 and/or lymph node involvement) or metastatic UCB. A total of 124 tumors were PD-L1 positive (+) (44.0%), either by a CPS ≥ 10 or an IC score of 2/3 or by both. Only 2.1% (6/282) of our study cohort received neoadjuvant cisplatin-based chemotherapy, with two patients showing PD-L1 positivity, while four patients were negative for PD-L1. Regarding adjuvant chemotherapy, 7.1% (20/282) of patients received adjuvant cisplatin-based chemotherapy, and such patients were equally distributed between both groups. In total, 162 patients developed metastases or a recurrence, of whom 38.3% (62/162) were PD-L1+, and 41.9% of the PD-L1+ patients received first-line (1-L) platinum-based chemotherapy (26/62). In PD-L1 negative (−) patients, 32.0% and 68% received ICIs or chemotherapy as 1-L treatment, respectively. In PD-L1+ patients, 41.9% and 58.1% received chemotherapy and ICIs as 1-L treatment. Following 1-L platinum-based chemotherapy, 84.5% and 97.1% of PD-L1+ and PD-L1− patients received ICIs as a 2-L treatment, respectively, while only 27.7% and 37.5% of PD-L1+ and PD-L1− patients received chemotherapy as a 2-L treatment after 1-L ICIs, respectively.

Table 1 shows the characteristics of all patients who received IHCs for their PD-L1 status. With 50.0% (62/124) for PD-L1+ and 63.3% (100/158) for PD-L1− patients, more than half of patients showed subsequent tumor recurrence or progressive disease. While local tumor recurrence and the nodal manifestation of metastases was most common for both groups, further sites of metastases included the lungs, skeleton, peritoneum and cerebrum. However, hepatic metastases were significantly more common in PD-L1− patients, with 24.0% (24/100) compared to only 6.5% (4/62) for PD-L1+ patients (*p* = 0.031). Comorbidities and *Eastern Cooperative Oncology Group* (ECOG) performance statuses were evenly distributed within and between our patient cohorts. While we report significantly more non-invasive tumors in the PD-L1− patient cohort, the majority of patients presented with locally advanced tumors, with 69.1% (112/162) ≥ pT3. There was no difference between groups in nodal or resection status.

### 3.2. TEAE

As 96.2% (156/162) of patients with progressive disease received ICIs either in 1-L or 2-L treatment, we aimed to evaluate the immune-related treatment-emergent adverse events (irTEAEs) in our patient cohorts (Table 2 shows any irTEAEs and their grade ≥ 2). We mostly encountered thyroiditis and consequent hypothyroidism with a need for hormone substitution, colitis, hepatitis and AEs involving the integument, ranging from simple pruritus, to a maculopapular rash, to bullous pemphigoid, especially in patients receiving pembrolizumab for >12 months. While we report a total of 96 (61.5%; 96/156) registered irTEAEs, we found no difference in the incidence or severity of irTEAEs when stratified by PD-L1 status (Table 3). All irTEAEs were treated according to the European Society for Medical Oncology (ESMO) guidelines [31].

### 3.3. PFS

We report no difference in the median PFS between patient cohorts, when divided by PD-L1 status (Table 4A). PFS showed no significant difference in 1-L or 2-L treatment, regardless of the treatment (chemotherapy vs. ICIs). Regardless of PD-L1 status, PFS was significantly longer for patients receiving ICIs as a 1-L treatment with a median PFS of 17.5 months (95% CI 9.2–25.8) versus 3.5 months (95% CI 2.6–4.4) for ICIs vs. chemotherapy, respectively (*p* < 0.001; Table 4B). However, there was no difference in 2-L treatment. Furthermore, we sought to evaluate the impact of PD-L1 status on treatment response. PD-L1+ patients receiving ICIs as a 1-L treatment had a significantly longer median PFS than PD-L1− patients (17.5 months [95% CI 11.0–24.1] vs. 3.4 [95% CI 2.9–3.9]; *p* < 0.001; Table 4C). However, PD-L1+ and PD-L1− patients receiving ICIs as a 1-L treatment also had a significantly longer median PFS than PD-L1− patients receiving chemotherapy (17.5 months [95% CI 11.0–24.1] and 6.3 months [95% CI 2.0–10.6] vs. 3.8 [95% CI 2.3–5.2]; *p* < 0.001 and *p* = 0.03, respectively; Table 4C).

### 3.4. OS

**Figure 2:**
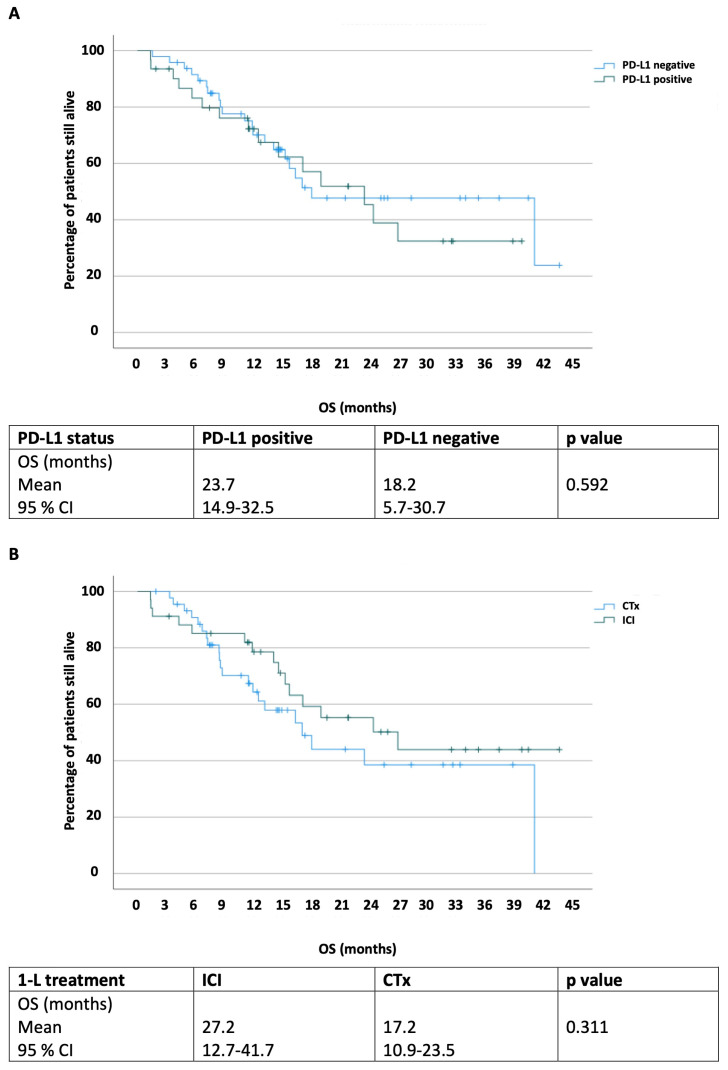
Overall survival stratified by PD-L1 status (**A**) and first-line treatment (**B**). All *p*-values < 0.05 are in bold script and are considered statistically significant. PD-L1 = programmed death 1 ligand 1; CI = confidence interval; CTx = chemotherapy; ICI = immune checkpoint inhibitor.

Regarding OS, we report no difference between groups, neither stratified by PD-L1 status (Figure 2A) nor by 1-L treatment (Figure 2B). The OS for PD-L1+ patients was 23.7 months (95% CI 14.9–32.5) versus 18.2 months (95% CI 5.7–30.7) for PD-L1- patients (*p* = 0.592). Patients receiving ICIs as a 1-L treatment showed a median OS of 27.2 months (95% CI 12.7–41.7) versus the 17.2 months (95% CI 10.9–23.5) for patients receiving chemotherapy (*p* = 0.311).

## 4. Discussion

The impact of PD-L1 status has been correlated with both favorable and unfavorable outcomes in various malignancies [32,33]. In UCB, PD-L1 expression has been associated with high-grade, advanced disease and worse outcomes. However, the overall impact of PD-L1 expression on disease progression and tumor prognosis, or even treatment response, remains controversial [34]. In addition, it has been suggested that PD-L1 expression may be associated with certain clinico-pathological features and clinical outcomes [19,20,34].

In 2019, Wen and colleagues aimed to evaluate the clinicopathological and prognostic value of PD-L1 in UCB in a meta-analysis, including 1819 patients from 11 studies [34]. Our analysis showed that the patient characteristics and tumor stage were evenly distributed between PD-L1− and PD-L1+ patients. While Wen et al. report a significant correlation between PD-L1 expression and ≥T2 tumor stage, we can confirm those results due to significantly more non-invasive tumors being present in the PD-L1− patient cohort. Corresponding to their analysis, we could show no correlation between nodal positivity and PD-L1 status (Table 1). Out of 11 eligible studies, nine studies reported results of OS toward PD-L1 expression.

While the meta-analysis of those studies showed the significant association of PD-L1 positivity and poor OS, we report no difference in OS and PD-L1 status. Our findings, thereby, correspond to the initial IMvigor210 trial, which lead to the approval in 2017 of PD-L1 inhibitor atezolizumab to be utilized by cisplatin-ineligible patients [12,35]. Treatment response was analyzed for the total cohort and according to PD-L1 expression on tumor-infiltrating immune cells, i.e., the IC score [35]. Responses occurred across all PD-L1 and poor prognostic factor subgroups. While the objective response rate of the total cohort was 23%, it rose to 28% in the PD-L1 subgroup IC2/3, to 24% in IC1/2/3, to 21% in IC1, and to 21% in IC0 patients. A median OS of 15.9 months was achieved for the overall study cohort, with the extent of PD-L1 expression showing no significant influence on the median OS [12].

The KEYNOTE 052 study led to the approval of pembrolizumab as a first-line therapy for cisplatin-unsuitable patients. Tumors with a positive PD-L1 staining (CPS ≥ 10) responded significantly better to the therapy with an objective response rate of 47%, a median PFS of 4.9 months and an OS of 18.5 months. For comparison, the OS in patients with CPS < 10 was 9.7 months and, thus, lower than in comparison with standard chemotherapy [36]. This, together with the preliminary results of Phase III trials in IMvigor130 and KEYNOTE-361 [26,27], lead to restricting 1-L approval to PD-L1-positive tumors (IC ≥ 5% for atezolizumab and CPS ≥ 10 for pembrolizumab) by the EMA and the FDA [24,25]. 

The determination of the prognostic and predictive value of a PD-L1 status is inconsistent in 1-L treatment, as seen in the IMvigor210 and KEYNOTE 052 trials, but also in the 2-L setting [24,25,37], as shown by Bellmunt testing pembrolizumab versus chemotherapy in patients with advanced UCB [37].

When dividing our patient cohorts by their PD-L1 status, we found no difference in PFS on 1-L or 2-L therapy, irrespective of treatment. We further stratified patients by detailed PD-L1 expression (Table 4A). We found significantly longer PFS on 1-L treatment, if patients were scored positively for both their IC score and CPS compared to patients with PD-L1 negative tumors. However, most PD-L1-negative patients received chemotherapy as a 1-L treatment (Figure 1). Further, in our cohort, ICIs (mostly pembrolizumab) induced a significantly longer PFS in 1-L compared to chemotherapy, but there might be biases in the observation (Table 4B). To reduce bias, we further stratified patients by PD-L1 status and treatment, leading to a most interesting observation: the PFS on 1-L treatment depended on whether patients received ICIs or chemotherapy (Table 4C). Patients receiving 1-L ICIs had a significantly longer PFS, regardless of their PD-L1 status. In PD-L1-negative patients, who could receive 1-L ICIs before PD-L1 status restriction, we found ICIs to be superior compared to chemotherapy, highlighting the weak predictive value of PD-L1 status in 1-L. This may be due to other potential targets of pembrolizumab, such as PD-L2 expression on the urothelium [38,39,40]. While little information exists regarding the expression of PD-L2 on urothelial bladder cancer as an alternative PD-1 ligand, there is mounting evidence for its constitutive expression as normal and or malignant in the urothelium [39]. Using immunohistochemistry, Dowell and colleagues found widespread expression of PD-L2 in UCB, albeit with reduced expression in muscle invasive disease. However, in contrast to the immunohistochemistry findings, expression was significantly increased in advanced disease, raising the question of its significance for treating patients with advanced UCB. While Dowell and colleagues propose a mechanism by which PD-L2 is cleaved from the cell surface in MIBCs, Yearley and colleagues found that the clinical response to pembrolizumab in patients with head and neck squamous cell carcinoma (HNSCC) with recurrent or metastatic disease may be related partly to the blockade of PD-1/PD-L2 interactions [38]. Thus, it can be concluded that therapy targeting both PD-1 ligands may provide a clinical benefit in patients expressing PD-L2 on the tumor surface. However, few reports exist on PD-L1-negative patients suffering from urothelial cancer with PD-L2 expression receiving immune checkpoint inhibition. George and colleagues present a case report, in which a single PD-L1-negative patient received pembrolizumab and experienced disease stability in upper-tract urothelial carcinoma (UTUC) for 20 months [40]. In this patient, RNA-seq analysis unveiled several biomarkers indicative of an active immune response and concurrent compensatory immune evasion. These included moderate PD-L1 levels alongside substantial PD-L2 expression. However, PD-L2 expression has become of research interest, as it may account for the effects of ICIs in PD-L1-negative patients [41]. Nonetheless, further evaluation and validation is warranted.

In our assessment of OS, we discerned no distinction between patients with a positive or a negative PD-L1 status, echoing the controversial findings reported in various studies [19,34,42]. Additionally, our findings indicate no variance in OS irrespective of 1-L treatment, highlighting the constrained predictive and prognostic utility of PD-L1 status. This inconsistency across multiple studies inherently restricts the clinical relevance of evaluating PD-L1 status [19,34,42]. Thus, our research focused solely on PD-L1 expression, which has become a center of attention for urologists over the past years, regarding the restriction of 1L immune checkpoint inhibitors in patients with UCB. However, MIBCs have a diverse mutation spectrum offering many more hypothetical therapeutical targets. Aiming for a comprehensive molecular characterization of muscle invasive bladder cancer, Robertson and colleagues identified five expression subtypes that may stratify the response to different treatments [43]. They distinguished a luminal-infiltrated subtype from other luminal subtypes with a strong expression of smooth muscle and myofibroblast gene signatures. This subtype has been documented to derive the greatest benefit from anti-PDL1 therapy and exhibited an intermediate 5-year survival rate, similar to other subtypes such as the basal-squamous and luminal [44]. These tumors demonstrated a heightened expression of various immune markers, notably CD274 (PD-L1) and PDCD1 (PD-1). These data offer promising insights into the urothelial cancer mutation burden, identifying subsets with an improved survival of subjects with a higher mutational burden. Although not yet validated, this should be recognized in ongoing clinical trials, including trials of immune checkpoint therapy. However, if those tumors correlated with our CPS- and IC-positive cohort showing a longer PFS, we can neither confirm nor deny it.

Given that most of our patients were treated with ICIs as either a 1-L or a 2-L therapy, our objective was to assess the irTEAEs within our patient cohorts. We found that PD-L1 expression in tumors did not influence the occurrence or severity of irTEAEs, indicating that this biomarker cannot serve as a predictor for immune-related adverse events.

The limitation of the biomarker PD-L1 was also observed in the recent EV-302 trial comparing the efficacy and safety of the ADC enfortumab vedotin (EV) and pembrolizumab with that of platinum-based chemotherapy in patients with previously untreated locally advanced or metastatic UC [45]. While the combination of the ADC and pembrolizumab will potentially revolutionize the 1-L treatment of UCB, the PD-L1 expression status measured by the CPS did not predict the response to the treatment, limiting its predictive value for this novel option.

Regarding perioperative cisplatin-based chemotherapy, we report no significant difference in PD-L1 expression among our patient cohort. While, as a tertiary referral center, patients’ eligibility for platinum-based chemotherapy may be limited by their age and comorbidities, our findings are in line with the previous work by Erlmeier and colleagues, who monitored PD-L1 expression on tumor cells during perioperative chemotherapy for urothelial cancer and evaluated their use as potential predictive markers for chemotherapy [46]. However, they conclude that the tumoral expression of PD-L1 in urothelial carcinoma does not seem to be largely influenced by chemotherapy. In our study, the proportion of patients receiving neoadjuvant chemotherapy was insubstantial, and patients were tested for PD-L1 status at the time of cystectomy. Thus, limiting the influence of adjuvant treatment on PD-L1 expression.

Our study, while insightful, has its limitations. The methodology involved the prospective enrollment of patients from a single tertiary referral center, introducing a potential selection bias, as evidenced by the limited number of patients receiving perioperative chemotherapy, as outlined above. Furthermore, the inclusion criteria spanned patients starting therapy from January 2018, which notably encompasses PD-L1-negative individuals receiving ICIs as a 1-L treatment, prior to the EMA’s restriction effective from June 2018. This inclusion could inadvertently skew our findings. The scope of our study, being confined to a single center with a relatively modest cohort size, inherently limits the robustness of our analysis. Furthermore, comparing our results with those from previous studies presents challenges, given the variance in methodologies applied to evaluate the PD-L1 expression in UCB.

However, and despite these constraints, we believe our findings contribute valuable insights into the conflicting value of PD-L1 expression and testing. The enhanced understanding of the futility and volatility of this biomarker underlines the need for better biomarkers for the prognosis of UCB and the response to agents targeting the immune checkpoint pathway.

## 5. Conclusions

Interestingly, our findings indicate that PFS is significantly dictated by the therapeutic approach chosen, with PD-L1 status playing a minimal role. Additionally, our data show uniform median OS outcomes across various treatments and irrespective of PD-L1 expression. Intriguingly, the responsiveness of PD-L1-negative patients to anti-PD-L1 therapy underscores the critical necessity for the identification of more accurate and robust biomarkers to predict responses to treatments targeting this pathway. The evolution of treatment strategies, particularly the anticipated integration of ICIs with ADCs in the 1-L treatment of bladder cancer, underscores this need even further.

## Figures and Tables

**Table 1:** Patient characteristics. Patients are divided into programmed death 1 ligand 1 (PD-L1) positive (+) and negative (−) groups, determined either by their immune cell (IC) score or their combined positive score (CPS). ECOG performance status = Eastern Cooperative Oncology Group. N/A = not applicable. All *p*-values < 0.05 are considered statistically significant and marked bold script.

Total Tested (n = 282)	PD-L1+ (n = 124)	PD-L1− (n = 158)	*p*-Value
**Age**			
Median	74	74	0.755
IQR	64–79	58–80	
**Sex**			
Male	76 (61.3%)	114 (72.2%)	0.436
Female	48 (38.7%)	44 (27.8%)	0.187
**PD-L1 status**			
IC 2/3	24 (19.3%)	N/A	
CPS ≥ 10	59 (47.6%)	N/A	
IC 2/3 + CPS ≥ 10	41 (33.1%)	N/A	
**Comorbidities**			
None	21 (16.9%)	47 (29.7%)	0.138
1	24 (19.4%)	35 (22.2%)	0.562
2	36 (29.0%)	35 (22.2%)	0.498
>2	43 (34.7%)	41 (26.0%)	0.322
**ECOG**			
0	40 (32.3%)	60 (38.0%)	0.417
1	52 (41.9%)	76 (48.1%)	0.502
≥2	32 (25.8%)	22 (13.9%)	0.177
**Progressive disease**	**62 (50.0%)**	**100 (63.3%)**	0.217
**Localization**			
Local	36 (58.1%)	96 (96.0%)	0.359
Nodal	50 (80.6%)	78 (78.0%)	0.548
Pulmonal	12 (19.4%)	20 (20.0%)	0.555
Hepatic	4 (6.5%)	24 (24.0%)	**0.031**
Bone	10 (16.1%)	16 (16.0%)	0.603
Peritoneal	6 (9.7%)	2 (2.0%)	0.165
Cerebral	2 (3.2%)	0 (0.0%)	0.392
**Staging**			
None-invasive tumors	0 (0.0%)	14 (14.0%)	**0.029**
T2	12 (19.4%)	24 (24.0%)	0.420
T3	28 (45.2%)	28 (28.0%)	0.091
T4	22 (35.5%)	34 (34.0%)	0.539
≥T2	62 (100%)	86 (86.0%)	**0.029**
**Nodal status**			
NX	14 (22.6%)	4 (4.0%)	0.112
N0	20 (32.3%)	28 (28.0%)	0.673
N1	16 (25.8%)	42 (42.0%)	0.107
N2	12 (19.4%)	18 (18.0%)	0.765
N3	0 (0.0%)	8 (8.0%)	0.138
N+	28 (45.2%)	68 (68.0%)	0.212
**Resection status**			
R0	48 (77.4%)	84 (84.0%)	0.664
R1	14 (22.6%)	16 (16.0%)	0.433

**Table 2:** Immune-related treatment-emergent adverse events (irTEAEs) including complication management. TRAEs included pruritus, thyroiditis, pneumonitis, colitis, skin rash, bullous pemphigoid, nephritis, hepatitis and hypophysitis. AEs were treated according to the European Society for Medical Oncology (ESMO) guidelines on irTEAEs.

TEAE; N (%) (n = 156)	Any Grade	Grade ≤ II	Grade III
**Overall TEAEs; N (%)**	96 (61.5%)	60 (37.0%)	16 (9.9%)
Thyroiditis	18 (11.5%)	12 (7.7%)	0
Colitis	16 (9.9%)	8 (5.1%)	2 (1.3%)
Hepatitis	12 (7.7%)	6 (3.8%)	4 (2.6%)
Fatigue	12 (7.7%)	6 (3.8%)	0
Pruritus	10 (6.4%)	6 (3.8%)	0
Rash, maculopapular	8 (5.1%)	6 (3.8%)	2 (1.3%)
Pneumonitis	6 (3.8%)	2 (1.3%)	4 (2.6%)
Nephritis	6 (3.8%)	4 (2.6%)	2 (1.3%)
Arthralgia	6 (3.8%)	2 (1.3%)	0
Hypophysitis	2 (1.3%)	0	2 (1.3%)

**Table 3:** Immune-related treatment-emergent adverse events (TEAEs). Patients are divided into programmed death 1 ligand 1 (PD-L1) positive (+) and negative (−) groups, determined either by their immune cell (IC) score or their combined positive score (CPS).

irTEAE	PD-L1+ (n = 58)	PD-L1− (n = 98)	*p*-Value
Overall TEAEs; N (%)	36 (62.6%)	60 (61.2%)	0.598
Grade I	8 (13.8%)	20 (20.4%)	0.338
Grade II	22 (37.9%)	30 (30.6%)	0.224
Grade III	6 (10.3%)	10 (10.2%)	0.767
Grade IV	0	0	

**Table 4:** Progression-free survival (PFS) stratified by PD-L1 status and line of treatment. All *p*-values < 0.05 are in bold script and are considered statistically significant. PD-L1 = programmed death 1 ligand 1; 1-L = first-line treatment; 2-L = second-line treatment; IC = immune cell; CPS = combined positive score; CI = confidence interval; CTx = chemotherapy; ICI = immune checkpoint inhibitor.

A						
PD-L1 Status		PD-L1 Negative	PD-L1 Positive	*p* Value		
PFS 1-L (months)				*p* = 0.162		
(n)		100	62			
Median		3.9	5.6			
95% CI		(3.2–4.6)	(0–17.0)			
PFS 2-L				*p* = 0.274		
(n)		78	32			
Median		4	6.5			
95% CI		(1.9–6.1)	(2.2–10.9)			
**PD-L1 Status**		**PD-L1 Negative**	**IC 2/3**	**CPS ≥ 10**	**IC 2/3 and** **CPS ≥ 10**	***p* Value**
PFS 1-L						*p* = 0.251 * *p* = 0.047
(n)		100	12	30	20	
Median		3.9	6	3.4	14.3 *	
95% CI		(3.2–4.6)	(1.4–10.5)	(2.5–4.4)	(1.6–29.5)	
PFS 2-L						*p* = 0.444
(n)		78	8	14	10	
Median		4	3.7	NR	6.5	
95% CI		(1.9–6.1)	(2.9–4.5)		(1.3–13.0)	
**B**						
**Treatment**		**CTx**	**ICI**	***p* Value**		
PFS 1-L				***p* < 0.001**		
(n)		94	68			
Median		3.5	17.5			
95% CI		(2.6–4.4)	(9.2–25.8)			
PFS 2-L				*p* = 0.671		
(n)		22	88			
Median		4.4	4			
95% CI		(3.7–5.1)	(1.3–6.7)			
**C**						
	**1-L Treatment**	**PFS 1-L**	**CTx**	**ICI**	***p* Value**	
**PD-L1 Status**						
**PD-L1 positive**		(n)	26	32	***p* < 0.001**	
		Median	3.4	17.5		
		95% CI	(2.9–3.9)	(11.0–24.1)		
**PD-L1 negative**		(n)	68	32	***p* = 0.03**	
		Median	3.8	6.3		
		95% CI	(2.3–5.2)	(2.0–10.6)		
***p* value**		***p* < 0.001**	*p* = 0.648	*p* = 0.327	*p* = 0.079	

* PD-L1 negative vs. IC2/3 and CPS ≥ 10.

## Data Availability

The data that support the findings of this study are available from the corresponding author upon reasonable request.
